# Neutron crystallography of photoactive yellow protein reveals unusual protonation state of Arg52 in the crystal

**DOI:** 10.1038/s41598-017-09718-9

**Published:** 2017-08-24

**Authors:** Kento Yonezawa, Nobutaka Shimizu, Kazuo Kurihara, Yoichi Yamazaki, Hironari Kamikubo, Mikio Kataoka

**Affiliations:** 1 Graduate School of Materials Science, Nara Institute of Science and Technology, 8916-5 Takayama, Ikoma, Nara, 630-0192 Japan; 2 Institute of Materials Structure Science, High Energy Accelerator Research Organization (KEK), 1-1 Oho, Tsukuba, Ibaraki, 305-0801 Japan; 3 National Institutes for Quantum and Radiological Science and Technology (QST), 2-4 Oaza- Shirakata, Tokai, Ibaraki, 319-1106 Japan; 4 Comprehensive Research Organization for Science and Society, Research Center for Neutron Science and Technology, 162-1 Shirakata, Tokai, Ibaraki, 319-1106 Japan

## Abstract

Because of its high pK_a_, arginine (Arg) is believed to be protonated even in the hydrophobic environment of the protein interior. However, our neutron crystallographic structure of photoactive yellow protein, a light sensor, demonstrated that Arg52 adopts an electrically neutral form. We also showed that the hydrogen bond between the chromophore and Glu46 is a so-called low barrier hydrogen bond (LBHB). Because both the neutral Arg and LBHB are unusual in proteins, these observations remain controversial. To validate our findings, we carried out neutron crystallographic analysis of the E46Q mutant of PYP. The resultant structure revealed that the proportion of the cationic form is higher in E46Q than in WT, although the cationic and neutral forms of Arg52 coexist in E46Q. These observations were confirmed by the occupancy of the deuterium atom bound to the N_**η**1_ atom combined with an alternative conformation of the N_(**η**2)_D_2_ group comprising sp^2^ hybridisation. Based on these results, we propose that the formation of the LBHB decreases the proton affinity of Arg52, stabilizing the neutral form in the crystal.

## Introduction

Although arginines (Args) in proteins are believed to be protonated even in the hydrophobic environment of protein interiors^1^, there are a few examples of electrically neutral Args in proteins^2, 3^. Arg52 in photoactive yellow protein (PYP), a light sensor, is one such exception^3^. Neutron crystallography of PYP revealed that one of the two deuterium atoms on either of the two ND_2_ groups in the guanidino group of Arg52 is lacked in the crystal state^3^. The existence of electrically neutral Arg and the mechanism underlying the decrease in pK_a_ remain under debate.

PYP is a light sensor responsible for negative phototaxis in the bacterium *Halorhodospira halophila* ^4, 5^. The protein’s chromophore, *p*-coumaric acid (pCA) bound to Cys69, isomerizes upon absorption of a photon, triggering the subsequent thermal reactions^6, 7^. Among them, proton transfer reactions within the hydrogen bond between pCA and Glu46 mediate structural changes of the protein moiety^8, 9^. Previously, it was thought that in the dark state of PYP, pCA adopts an anionic form whereas carboxyl group of Glu46 is protonated, and the proton within the hydrogen bond is transferred from Glu46 to pCA during the photoreaction^10^. Because pCA is buried in the hydrophobic environment of the protein interior, the isolated negative charge of the ionic form of pCA should require a counter-cation. A positive charge on Arg52 at a distance of 6.34 Å from pCA was proposed as a candidate for the counterion^11^.

Although recent progress in X-ray crystallography has enabled us to determine positions of hydrogen atoms, several limitations remain, including the requirement that crystals be cryo-cooled to 100 K to control radiation damage and obtain sub-atomic resolution data. In addition, highly polarised H atoms involved in hydrogen bonds are barely visible, even at sub-atomic resolution. Therefore, neutron crystallography can be considered the sole method for determining the positions of mobile or highly polarised hydrogen atoms even at room temperature^12^. Previously, we carried out neutron crystallographic analysis of PYP at room temperature to investigate the highly polarised hydrogen atoms involved in the hydrogen-bonding network near the chromophore. The results revealed that pCA and Glu46 engage in a special type of hydrogen bond, termed a low-barrier hydrogen bond (LBHB), in which the observed hydrogen atom is located near the centre position between pCA and Glu46^3, 13, 14^. Furthermore, the Arg52 is deprotonated to the electrically neutral form, and thus cannot play a role as a counterion in the crystal state. In this system, the negative charge would be delocalized along both pCA and Glu46, conjugated by the LBHB, allowing the negative charge to exist even in the protein interior. These findings inspired several researchers to attempt to reproduce these unusual phenomena in theoretical studies. Saito and Ishikita performed QM/MM calculations, but could not reproduce the LBHB in PYP^15^. It should be noted that in contrast to the reported crystal structures, their calculation assumed an energy-optimized structure as the supposed solution structure. Furthermore, Arg52 was assumed to be in the cationic form, as usual. Hirano and Sato also investigated the migration potential of the proton between pCA and Glu46 using the ONIOM method^16^. In their calculation, the geometry of the protein moiety was fixed to that of the crystal structure; in addition, they assumed that Arg52 was in the electrically neutral form, as reported in the neutron crystallographic analysis. The resultant migration potential was still asymmetric, but the energy gap between the two minima was smaller than that calculated by Saito and Ishikita, such that barrier between the two minima is suitable for realizing the LBHB. These results, along with those of other theoretical studies^17–22^, implied that the hydrogen bond between pCA and Glu46 depends on the structure used for calculation, and might be influenced by the charge state of the surrounding residues. Nadal-Ferret *et al*. examined the relationship using the QM/MM method based on two distinct structures of PYP: one is the crystal structure and the other is a presumed solution structure obtained from energy minimization using molecular dynamics simulation^23^. They postulated that the crystal structure retains the neutral Arg, whereas the Arg in the solution structure should adopt a cationic form due to exposure of the Arg by fluctuation of the structure. They concluded that LBHB can exist in the crystal structure, whereas in solution the proton prefers to localize near Glu46, resulting in an ionic hydrogen bond.

In this study, we sought to investigate the proton affinity of Arg52, perturbed by the hydrogen bond between pCA and Glu46, in the crystal state. To this end, we performed X-ray and neutron joint crystallographic analysis on the E46Q mutant, and compared the structure of Arg52 with previous results obtained using the wild-type protein (WT). Replacement of Glu46 with Gln resulted in conversion of the LBHB in WT into an ordinary hydrogen bond. Although we observed no obvious nuclear density of one of the two positions on N_η1_ of Arg52 in WT, the nuclear density map of E46Q showed nuclear density of the two deuterium atoms on N_η1_ even at the same contour level as in WT, indicating that Arg52 is more highly protonated in E46Q than in WT. In fact, the occupancies of the deuterium atom bound to the N_η1_ atom combined with an alternative conformation of the N_(η2)_D_2_ group comprising sp^2^ hybridisation are estimated to be 24% for WT and 67% for E46Q. Based on these observations, we conclude that the hydrogen bond near the chromophore influences the pK_a_ of Arg52 in the crystal state of PYP.

## Results

### Hydrogen bonds near the chromophore in WT and E46Q

**Figure 1:**
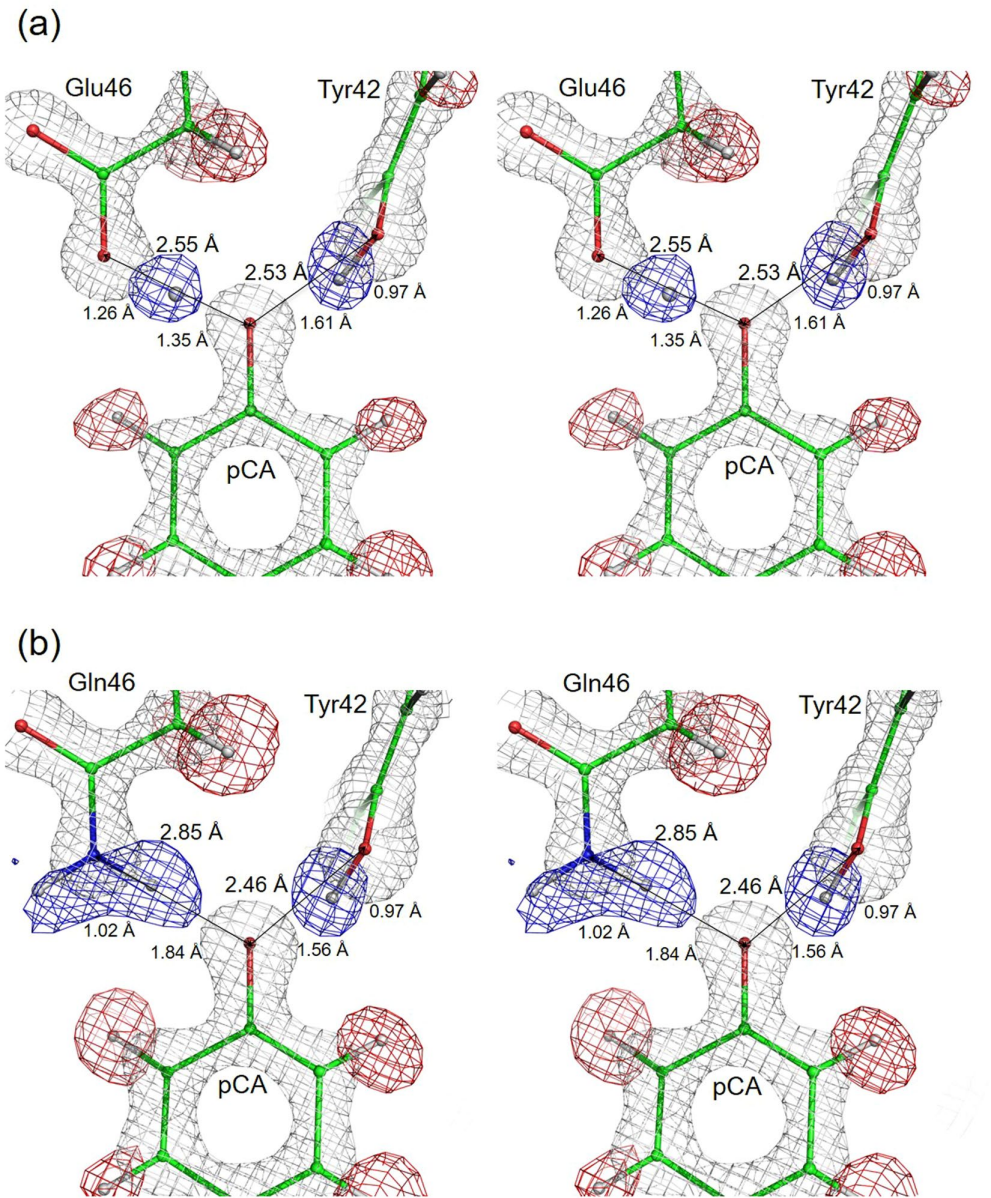
Hydrogen bonds near the chromophore, including hydrogen and deuterium atoms. Nuclear and electron density maps superposed on the structural models of WT (**a**) and E46Q (**b**). Blue meshes represent positive nuclear density of the Fo–Fc maps omitting each deuterium atom involved in the two hydrogen bonds, contoured at 5.0σ. Red mesh represents the Fo–Fc nuclear density maps, contoured at −5.0σ, omitting the hydrogen atoms. 2Fo–Fc electron density maps of heavy atoms contoured at 3.5σ (**a**) and 3.0σ (**b**) are also shown.

To investigate the effect of the hydrogen bond between pCA and Glu46 on the protonation state of Arg52, we carried out neutron and X-ray diffraction experiments on E46Q at room temperature. Neutron beams can be strongly diffracted, even by small atoms such as hydrogen/deuterium atoms, at the same level as the heavy atoms that comprise proteins. Consequently, a neutron structure contains twice as many atoms as a comparable X-ray structure. This situation can sometimes be problematic, as the data-to-parameter ratio is low. In order to overcome this challenge, in our previous neutron crystallographic analysis of WT, we also collected both neutron and X-ray diffraction images using the same crystal, and then separately determined the positions of the heavy atoms and hydrogen/deuterium atoms from the X-ray and neutron data, respectively^3^. New structural refinement programs have recently been developed that enable us to refine structure models using neutron and X-ray data simultaneously, yielding a substantial improvement in the accuracy of structure determination^12, 24–26^. Therefore, we carried out the X-ray and neutron joint analysis of E46Q, as well as WT, using this modern form of joint analysis. The backbone structure of E46Q superposes well on that of the WT (r.m.s.d of the all C_α_ atoms < 0.08 Å), consistent with the previous analysis^27, 28^. Although the backbone structure of E46Q, except for the protein moiety neighbouring pCA, is almost identical to that of WT, the hydrogen bond pCA–Gln46 was perturbed upon replacement of Glu46 with Gln. Figure 1 shows the nuclear and electron density maps of WT and E46Q superposed on the structural models near pCA; the Fo–Fc nuclear density maps (omitting deuterium and hydrogen atoms) are represented in blue and red for WT and E46Q, respectively, and the 2Fo–Fc electron density maps are coloured in grey. Representative interatomic distances are shown in the figure and also summarised in the Table S1. The joint analysis upon WT improved the R-factor (R_free_) from 19.2 (21.9) to 16.2 (20.8) in comparison with the previous analysis^3^ (Table S3). The previous and current structures of WT are almost identical within r.m.s.d. of 0.05 Å. The nuclear density maps of E46Q show that the ND_2_ group of Gln46 is oriented toward the phenolic oxygen of pCA to form the hydrogen bond, as predicted previously^27, 28^. In E46Q, the hydrogen bond length between Gln46 and pCA increases to 2.85 ± 0.04 Å (from 2.55 ± 0.03 Å in the WT). Although the deuterium atom is located near the centre position between Glu46 and pCA in WT, the deuterium atom in E46Q is covalently bound to Gln46, with a bond length of 1.02 Å. The hydrogen bond between pCA and Tyr42 was also slightly influenced by the mutation: the hydrogen bond length in E46Q was decreased by 0.07 Å (from 2.53 ± 0.03 Å in WT to 2.46 ± 0.04 Å in E46Q). These results confirm that the LBHB in WT is converted into an ordinary hydrogen bond by the E46Q mutation.

### Nuclear density maps of Arg52 in WT and E46Q

**Figure 2:**
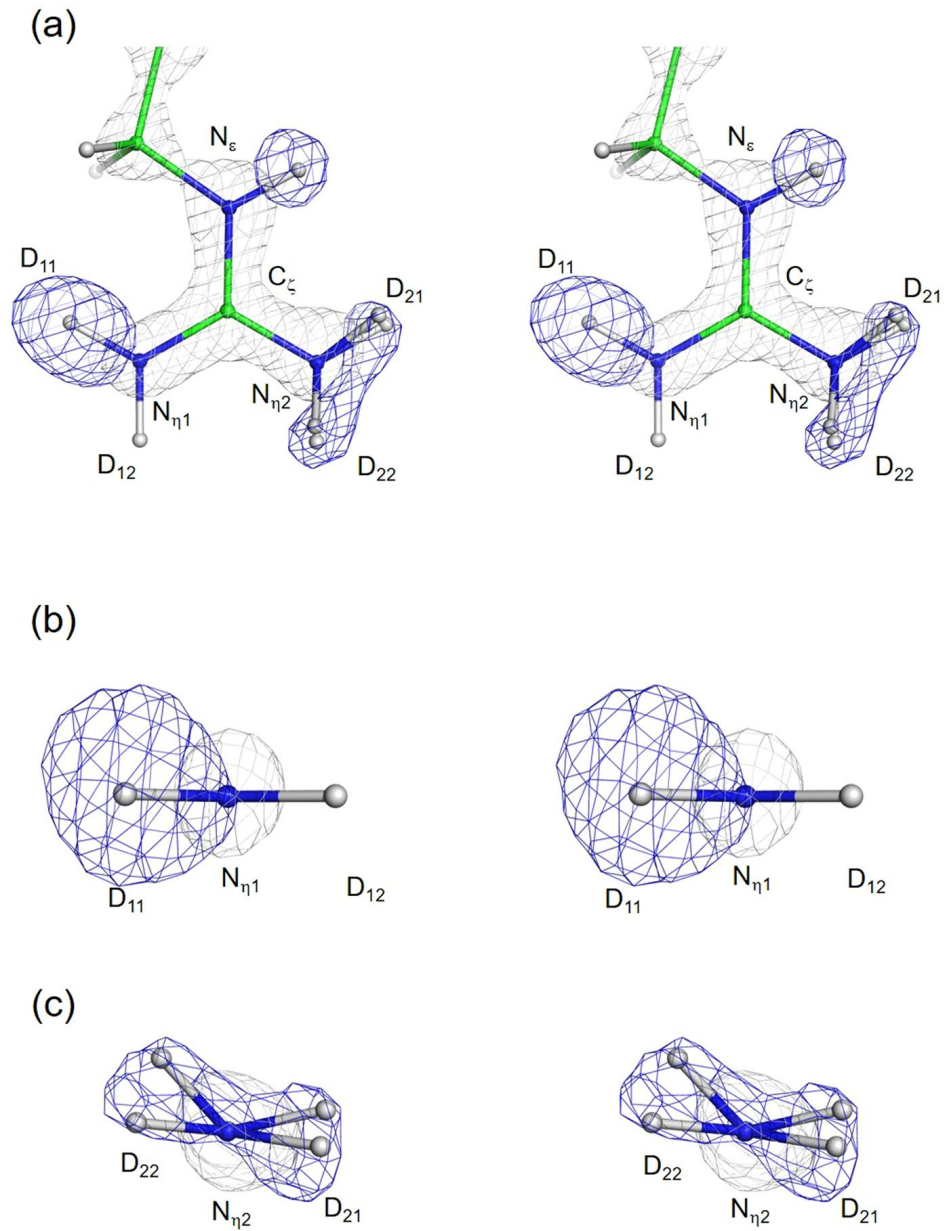
Nuclear density maps of the guanidino group of in WT. Top view of the guanidino group (**a**), and the N_η1_ side (**b**) and N_η2_ side (**c**) of the ND_2_ group. Fo–Fc nuclear density maps omitting the deuterium atoms and 2Fo–Fc electron density map of heavy atoms of Arg52 are shown as blue and gray mesh, respectively. Blue meshes represent nuclear density at contour levels of 45% (5.1σ) of the maximum peak height of D_11_. 2Fo–Fc electron density maps are contoured at 3.5σ. Refined structures of the N_(η2)_D_2_ group exhibiting an sp^2^ and an sp^3^ hybridisation are superposed.

In a previous study, we reported that the nuclear density of one of the deuterium atoms on N_η1_ of Arg52 in WT was not observed at contour level of the half-maximum peak height of the other deuterium atom^3^. This implies that Arg52 in WT adopts an electrically neutral form. Although the cationic and neutral forms should exhibit different hybridisations, sp^2^ and sp^3^, in the NH_2_ group in the guanidino group, the detailed structure of the NH_2_ group has not been revealed. Figure 2(a) shows the Fo–Fc nuclear density map omitting the deuterium atoms in WT (blue mesh), which was calculated from the re-refined WT structure by the joint analysis. The major characteristics of the Fo–Fc map reported in the previous paper are maintained in the current analysis^3^. One of the two deuterium atoms (D_12_) on the N_η1_ atom cannot be seen at the 45% of the maximum contour level of the other deuterium atom (D_11_), as reported previously. Assuming that Arg adopts an electrically neutral form, the N_(η2)_D_2_ group of the guanidino group should exhibit an sp^3^ hybridisation. Careful comparison of the nuclear density maps between the N_(η1)_D_2_ group in Fig. 2(b) and the N_(η2)_D_2_ group in Fig. 2(c) revealed that while the deuterium atom in the N_(η1)_D_2_ group is aligned with the plane defined by the heavy atoms (CN_3_) of the guanidino group, those of the N_(η2)_D_2_ group are distributed slightly above the plane. Two possible structures of the N_(η2)_D_2_ of the hybridisations of sp^2^ and sp^3^ are superposed onto the nuclear density map in Fig. 2. The deuterium atoms in the structure of sp^3^ can superpose onto the nuclear density, indicating that the major species of the N_(η2)_D_2_ group adopts the sp^3^ structure, which should be observed only in the electrically neutral form.

**Figure 3:**
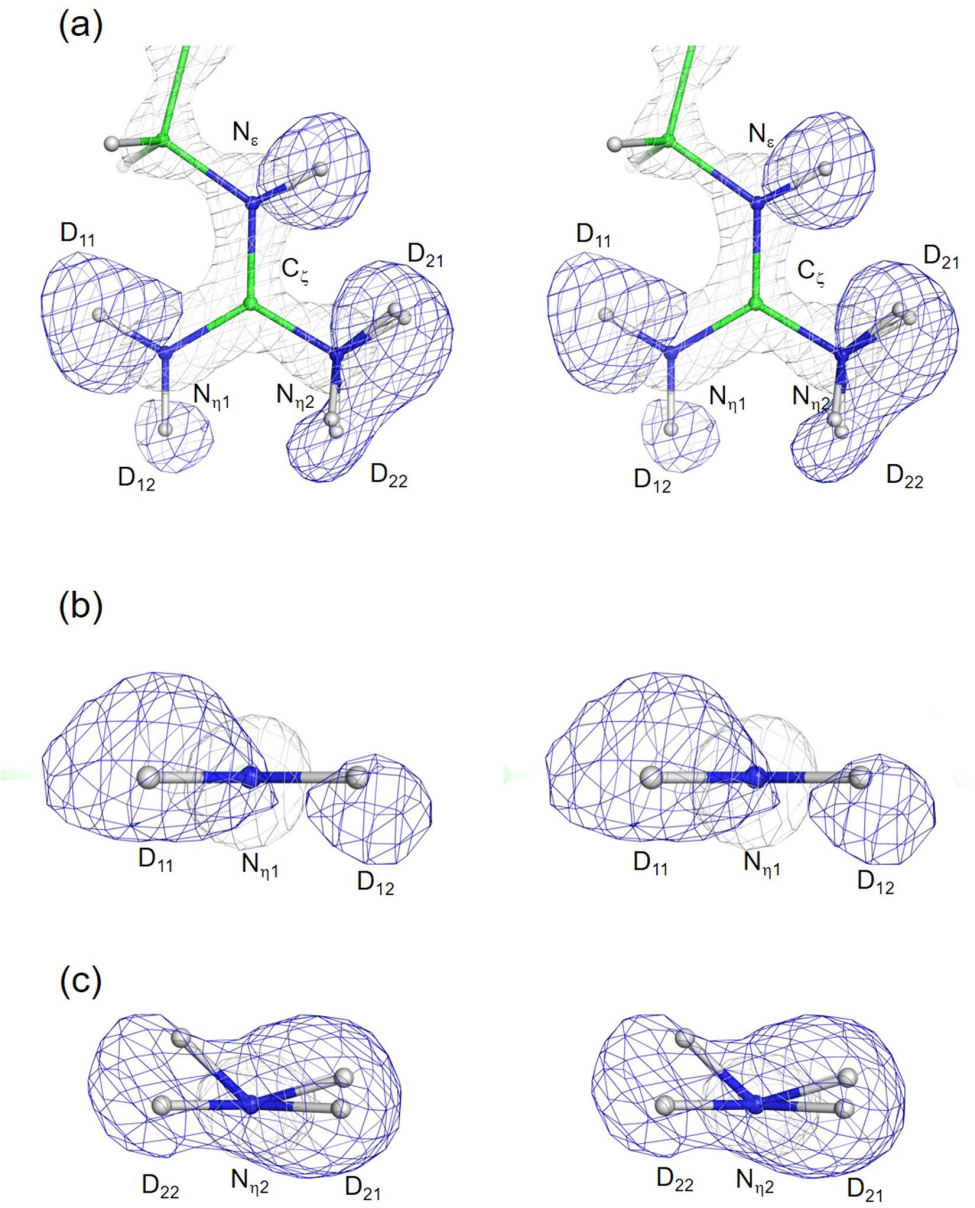
Nuclear density maps of the guanidino group of in E46Q. Top view of the guanidino group (**a**), and the N_η1_ side (**b**) and N_η2_ side (**c**) of the ND_2_ group. The Fo–Fc nuclear density maps omitting the deuterium atoms and 2Fo–Fc electron density map of heavy atoms of Arg52 are shown as blue and gray mesh, respectively. Blue meshes represent nuclear density at contour levels of 45% (4.5σ) of the maximum peak height of D_11_. 2Fo–Fc electron density maps are contoured at 3.0σ. Refined structures of the N_(η2)_D_2_ group exhibiting an sp^2^ and an sp^3^ hybridisation are superposed.

Nuclear density maps of Arg52 in E46Q are shown in Fig. 3. In E46Q, obvious nuclear density appears at the position of the D_12_ atom at the 45% of the maximum contour level of D_11_ [Fig. 3(a)], indicating that it contains a larger proportion of the cationic form than WT. Comparison of the nuclear density of the N_(η2)_D_2_ group between E46Q [Fig. 3(c)] and WT [Fig. 2(c)] revealed that the nuclear density distribution of E46Q is shifted toward the guanidino plane in comparison with WT. Considering that a larger proportion of the cationic form of Arg is accumulated in E46Q than in WT, as shown in Fig. 3(a), the sp^2^ structure of the cationic form of Arg can be included as a major species in the Fo–Fc maps [Fig. 3 (c)]. It should be noted that the nuclear density of the D_12_ atom is still smaller than that of D_11_, suggesting that Arg52 in E46Q also contains a smaller proportion of the neutral form, which is the major form in WT. In fact, the nuclear density maps of the N_(η2)_D_2_ group in E46Q are distinguishable from those of the N_(η1)_D_2_ group. Although the nuclear densities of the two deuterium atoms on the N_η1_ atom are well resolved in E46Q, those on the N_η2_ atom overlap in E46Q, suggesting the existence of sp^3^ hybridisation.

### Decomposition of the sp^2^ and sp^3^ hybridisations in the N_(η2)_D_2_ group

Figure S1 shows the nuclear density maps of the guanidino group of Arg52 in WT [(a)–(c)] and E46Q [(d)–(f)] at various contour levels. The most prominent peak can be observed at the position of D_11_ in both WT and E46Q. The occupancies of the D_11_ atoms (represented by an arrow in Figure S1) were close to 1. The contour levels are set to be 30, 40, and 50% of the peak height of D_11_ in Figure S1. As shown in Figs 2 and 3, the nuclear density of the D_12_ atom in E46Q can be observed at a contour level of 50%, but not in WT. However, when the contour level is decreased to 30%, close to the noise level, nuclear density appears even in WT, suggesting that a small subpopulation of Arg52 in WT adopts the cationic form [Figure S1(c)].

**Figure 4:**
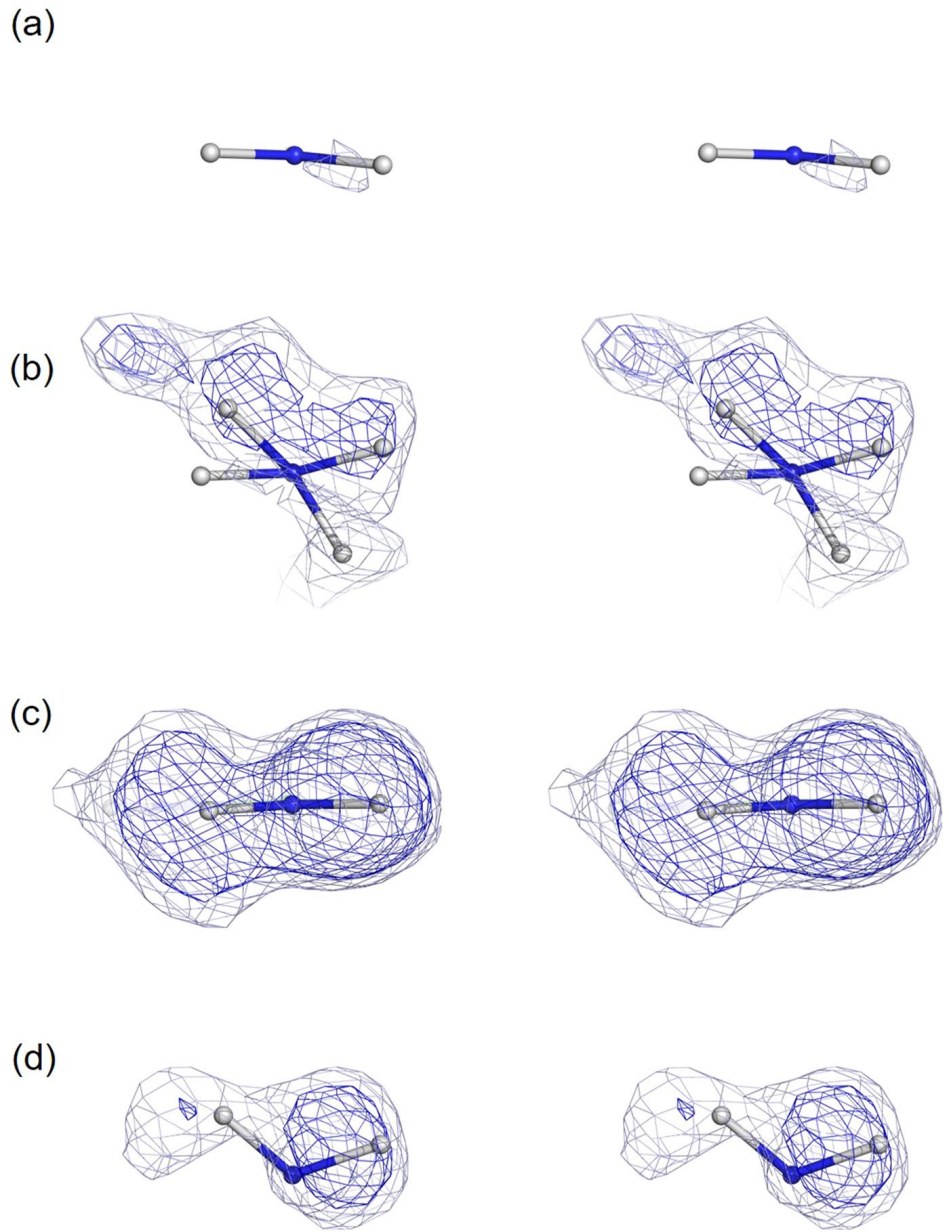
Decomposition of the sp^2^ and sp^3^ structures in the N_(η2)_D_2_ group. Refined structures and Fo–Fc nuclear density maps omitting the deuterium atoms of each N_(η2)_D_2_ group of WT (**a**,**b**) and those of E46Q (**c**,**d**). Nuclear density maps omitting the sp^2^ structure are shown in (**a**) and (**c**), and those omitting the sp^3^ structure are shown in (**b**) and (**d**). Blue and light blue meshes represent nuclear density contoured at 3.0σ and 2.0σ.

Reflecting the difference in the major species between WT and E46Q, the shapes of the nuclear density of the N_(η2)_D_2_ group are distinguishable. Assuming the coexistence of the cationic and neutral forms, the molar fraction of the sp^2^ hybridisation in the N_(η2)_D_2_ group should be identical to the occupancy of D_12_. Through the detailed structure refinement of Arg52 described in the Methods section, we assumed alternative groups consisting of D_12_ on N_η1_ and the two deuterium atoms of the sp^2^ hybridisation in the N_(η2)_D_2_ group, or the two deuterium atoms of the sp^3^ hybridisation in the N_(η2)_D_2_ group (Fig. 4). In the case of WT, the sp^3^ hybridisation in the N_(η2)_D_2_ group adopts two orientations (upward and downward) relative to the guanidino plane [Fig. 4(b)]. After several cycles of occupancy refinement of the alternative structures, and *B*-factor and position refinement of individual deuterium atoms, we obtained structure models of sp^2^ and sp^3^ of WT [Fig. 4(a) and (b)] and E46Q [Fig. 4(c) and (d)], as well as the occupancies of the alternative sets of the sp^2^ and sp^3^ hybridisations. The obtained parameters are summarised in Table S2. The occupancies of the cationic form containing D_12_ and the two deuterium atoms of the sp^2^ hybridisation in the N_(η2)_D_2_ group in WT and E46Q are 24% and 67%, respectively (Table S2). In addition, the Fo–Fc maps omitting the structural model for either sp^2^ or sp^3^ hybridisations are well superposed. Prominent nuclear densities are observed in the Fo–Fc maps omitting the sp^3^ model for WT [Fig. 4(b)] and the sp^2^ model for E46Q [Fig. 4(c)] even at 3 σ. Decreasing the contour level to 2.0 σ, the Fo–Fc maps omitting the sp^2^ model for WT [Fig. 4(a)] and the sp^3^ model for E46Q [Fig. 4(d)] also appear. In the case of WT, nuclear density was observed in the Fo–Fc maps omitting the sp^3^ model. Figure 4(b) shows the Fo–Fc maps omitting both of the two alternative sp^3^ models. The upward sp^3^ model with the higher occupancy (40%) was especially well superposed on the nuclear density [Fig. 4(b)]. The nuclear density of the Fo–Fc map omitting the sp^2^ model in Fig. 4(a) was less prominent, partly due to the smaller fraction (24%) in comparison with the sp^3^ hybridisation, but it nonetheless aligned with the model of sp^2^. On the other hand, clear nuclear density maps of both sp^2^ and sp^3^ species could be observed in E46Q. By omitting the structure of sp^2^ [Fig﻿﻿. 4(c)], the sp^2^ model can be superposed on the nuclear density of the deuterium atoms. The nuclear density is well aligned on the guanidino plane, which is characteristic of sp^2^ hybridisation. Furthermore, even the sp^3^ model of the minor species (33%) in E46Q, in which the nuclear density map are distributed above the guanidino plane, was resolved. Based on these results, we conclude that the guanidino groups in WT and E46Q contain both the cationic and neutral forms, and that the proportion of the cationic form is increased by replacement of Glu46 with Gln.

## Discussion

We previously reported that Arg52 of PYP WT in the crystal state can adopt a neutral form. Considering the high pK_a_ of Arg, the idea that Arg52 is electrically neutral is difficult to accept, and the issue remains a matter for debate^15–23^. In this study, we performed X-ray and neutron joint crystallographic analysis on E46Q to reveal the protonation state of Arg52. The results revealed that Arg52 can also adopt the neutral form in E46Q. This observation strongly confirms the existence of the neutral form of Arg in the crystal state of PYP. Furthermore, through detailed structural analysis, we revealed that Arg52 in both WT and E46Q is in equilibrium between the cationic and neutral forms. It should be emphasised that this equilibrium is shifted toward the cationic form by the mutation, which causes the molar fraction of the cationic form to increase from 24% to 67%. The crystal structures of WT and E46Q do not substantially differ except at the hydrogen bond between pCA and Glu46. The mutation increases the length of this hydrogen bond from 2.55 ± 0.03 to 2.85 ± 0.04 Å, resulting in disruption of the LBHB. Based on these results, we propose that the hydrogen bond mediates the proton affinity of Arg52. Recent theoretical studies suggested that the hydrogen bond between pCA and Glu46 is highly influenced by the protonation state of Arg52^16, 20–23^, i.e., that the charge on Arg52 electrostatically perturbs the molecular orbital of pCA from a distance of 6.34 Å. Based on our previous neutron crystallographic analysis of WT, we proposed that LBHB between pCA and Glu46 combines the two π-conjugated systems of pCA and the carboxyl group on Glu46, resulting in charge delocalization along the extended π-conjugated system^3^. The conversion of the LBHB into an ordinary hydrogen bond upon mutation would disrupt the extended π-conjugated systems, resulting in localization of the charge on pCA. Therefore, we propose that the increase in the proton affinity of Arg52 in E46Q arises from the increase in the charge density on pCA resulting from the disruption of the LBHB. The interdependency between the hydrogen bond between pCA and Glu46 and the proton affinity of Arg52 is expected to play a role in the photoreaction of PYP. A recent time-resolved X-ray crystallographic analysis revealed that the disruption of the LBHB is the first molecular event to occur after light absorption by the chromophore during the photoreaction of PYP^29^. The hydrogen bond length increases from 2.56 Å to 2.61 Å during the formation of the I_1_ intermediate. Arg52 slightly moves away from pCA at the I_1_ intermediate, and ultimately undergoes large structural change in the I_2_ intermediate, in which Arg52 is exposed to solvent^29, 30^. The alteration of the protonation state of Arg52 during the photoreaction should be examined in future studies, as the structural change observed at I_1_ and I_2_ might be closely related to the coupling of the disruption of the LBHB, which modulates the proton affinity of Arg52.

## Methods

### Preparation and crystallization of PYP for crystallographic analysis

The E46Q mutant of PYP were expressed and purified by using previous methods^3, 31^. PYP was expressed using the pET system in *Escherichia coli* BL21(DE3) and reconstituted with pCA anhydride in 4 M urea buffer^32^. Urea and other additive salts were removed by dialysis, and then PYP was purified by DEAE–Sepharose column chromatography and size-exclusion chromatography. Purification was repeated several times until the optical purity index (Abs_280_/Abs_λ460_) was less than 0.38. Large crystals of E46Q were obtained using the method reported by Yamaguchi *et al*.^31^. The initial protein solution was prepared at 24 mg/ml PYP in 20 mM sodium phosphate buffer containing 2.2 M ammonium sulfate and 1 M sodium chloride, and then equilibrated against reservoir solution containing 2.5 M ammonium sulfate and 1.1–1.2 M sodium chloride at 295 K. The hydrogen atoms of crystals of E46Q in this paper were exchanged with deuterium atoms. H/D exchange was performed by ultrafiltration several times.

### Data collection, processing, and refinement of X-ray and neutron crystallographic analysis

Both of X-ray and neutron diffraction experiments were performed at room temperature. A crystal (5.1 × 1.2 × 0.7 mm^3^) was trapped in a quartz capillary. The capillary contained reservoir solution at the bottom to prevent the crystal from drying. Neutron diffraction experiments up to 1.50 Å were also carried out using a neutron single-crystal diffractometer (BIX-4) at the JRR-3 reactor, Ibaraki, Japan^33^. The wavelength was set to 2.6 Å. The camera distance was 200 mm. Data were collected using step scans (0.3°/step), and 211 frames were recorded with an exposure time of 4 hours for each frame. Datasets were processed using DENZO and SCALEPACK^34^. X-ray diffraction data of E46Q were collected using the same crystal used in the neutron diffraction experiment at BL-6A of the Photon Factory, Ibaraki, Japan. Diffraction data up to 1.30 Å resolution were recorded using a Quantum 4 R detector over a rotation of 180° with an oscillation step of 1.0°. The wavelength was set at 0.98 Å. Camera distance and exposure time were 84.4 mm and 1 sec, respectively. Because the whole crystal was exposed to the neutron beam, we collected diffraction data at five different points along the longer axis of the crystal and averaged them, as appropriate for joint analysis of neutron and X-ray data. The collected X-ray data were integrated and merged with DENZO and SCALEPACK^34^.

Although the neutron structure of WT was reported previously, in that study the X-ray and neutron data were used separately to refine the heavy atoms and hydrogen/deuterium atoms, respectively^3^. Here, we refined the structure of WT as well as E46Q using a newly developed program (Phenix) that allows simultaneous refinement of X-ray and neutron structures^25^. Initial phases were determined by molecular replacement using previously reported structures (PDB; 1OTA for E46Q and PDB; 2ZOH for WT)^3, 27^. Model building was carried out with Coot and XtalView^35, 36^. Subsequent structure refinements were performed with Phenix^25, 26^. The X-ray structures containing only heavy atoms were first refined using the only X-ray data. After adding hydrogen/deuterium atoms to the obtained X-ray structure, the positions and the B-factors of the hydrogen/deuterium atoms were refined using the neutron data. The resultant structures of WT and E46Q were subject to the neutron and X-ray joint analysis using Phenix. We carried out several series of refinements of the B-factor and the positions of the heavy atoms and the hydrogen/deuterium atoms, continuing until those values were well converged. After fixing the positions of the heavy atoms, we refined the B-factor and the positions of the hydrogen/deuterium atoms using only the neutron data, again, in which the B-factors of the heavy atoms were also refined. Finally, we determined the fractions of exchangeable hydrogen/deuterium atoms. The peak heights in the Fo–Fc map of the deuterium atoms on the guanidino group, except for D_12_, we focus on in this paper, ranged from 6 σ to 11 σ. We searched for nuclear density peaks in the Fo–Fc map comparable to those on the guanidino group, and found four peaks in WT (Figure S2) and 11 in E46Q (Figure S3) above +6 σ or below −6 σ. Some of the peaks disappeared upon addition of alternative structures, whereas others could not be improved (see Figures S2 and S3). The origin of the rest artificial peaks remains unclear, but because these regions are distant from Arg52, they are not serious issues in the current analysis.

Subsequently, we performed further structural refinement of Arg52 to evaluate the molar ratio of the cationic and neutral forms of Arg52 and decompose the sp^2^ and sp^3^ structures of the N_(η2)_D_2_ group of WT and E46Q. If the neutral and cationic forms of Arg52 coexist, the molar ratio of the sp^2^ structure in the N_(η2)_D_2_ group is identical to the occupancy of the D_12_ atom. To decompose the nuclear density map responsible for sp^2^ or sp^3^, we assumed alternative groups consisting of D_12_ on N_η1_ and the two deuterium atoms of the sp^2^ hybridisation in the N_(η2)_D_2_ group, and the two deuterium atoms of the sp^3^ hybridisation in the N_(η2)_D_2_ group, in which the deuterium atoms in each group have an occupancy reflecting the molar ratio of the sp^2^ and sp^3^ structures. Through this refinement, we found that the sp^3^ hybridisation in the N_(η2)_D_2_ group in WT exhibits two distinct orientations in which the deuterium atoms are distributed above or below the guanidino plane. The three alternative structures for WT and the two alternative structures for E46Q are shown in Fig. 4. The occupancies of these groups were refined simultaneously. Although the parameter files for the cationic form of Arg are available in Phenix, those of the neutral form are not. We built the neutral form of Arg by replacing the sp^2^ structure of the N_(η2)_D_2_ group, derived from the cationic form, with the sp^3^ structure. The parameters defining the sp^3^ form were obtained from SwissParam:^37^ the bond length of D–N_η2_ was 1.0 Å, and the angles of D_21_–N_η2_–D_22_ and C_ζ_–N_η2_–D were 109.2° and 108.8°, respectively. The structural restriction for the dihedral and improper angle was weakened to enable the N_(η2)_D_2_ group of sp^3^ to rotate freely around the C_ζ_–N_η2_ bond. In the initial model of sp^3^ applied to the refinement, two deuterium atoms (D_21_ and D_22_) were parallel to the guanidino plane. The alternative groups of the sp^2^ and sp^3^ forms were refined by an energy-minimisation procedure, and then the *B*-factors of the individual deuterium atoms were also alternately refined by B-individual in Phenix. Refinements were performed until convergence. The statistics of data collection of WT and E46Q are provided in Table S3. The resultant structural models of sp^2^ and sp^3^ are superposed in Fig. 4. All structural models were drawn using PyMOL^38^.

Concerning the validity of the hydrogen bond lengths shown in Table S1, we calculated the estimated standard deviation (e.s.d) values of the distances by using ShelxL2013^39, 40^. In general, to obtain the e.s.d values, sub-atomic resolution data should be required. Applying the geometry constraints (AFIX) to the hydrogen/deuterium atoms involved in the refined structure, the current X-ray data at the resolution of 1.3 Å allowed us to calculate the e.s.d values of the hydrogen bond lengths between pCA and Glx46, and pCA and Tyr42 (Table S1)^40^.

### Data deposition

Model coordinates and structural factors have been deposited in the Protein Data Bank as entries 5GX9 (Neutron structure of E46Q).

## Electronic supplementary material

Supplementary Information

## References

[1] Fitch CA, Platzer G, Okon M, Garcia-Moreno BE, McIntosh LP (2015). Arginine: Its pKa value revisited. Protein Sci..

[2] Xiao Y, Hutson MS, Belenky M, Herzfeld J, Braiman MS (2004). Role of arginine-82 in fast proton release during the bacteriorhodopsin photocycle: A time-resolved FT-IR study of purple membranes containing 15N-labeled arginine. Biochemistry.

[3] Yamaguchi S (2009). Low-barrier hydrogen bond in photoactive yellow protein. Proc. Natl. Acad. Sci. USA..

[4] Meyer TE (1985). Isolation and characterization of soluble cytochromes, ferredoxins and other chromophoric proteins from the halophilic phototrophic bacterium Ectothiorhodospira halophila. Biochim. Biophys. Acta.

[5] Sprenger WW, Hoff WD, Armitage JP, Hellingwerf KJ (1993). The Eubacterium Ectothiorhodospira-halophila Is negatively phototactic, with a wavelength dependence that fits the absorption-spectrum of the photoactive yellow protein. J. Bacteriol..

[6] Imamoto Y, Kataoka M, Tokunaga F (1996). Photoreaction cycle of photoactive yellow protein from Ectothiorhodospira halophila studied by low-temperature spectroscopy. Biochemistry.

[7] Imamoto Y (2001). Low temperature Fourier transform infrared spectroscopy of photoactive yellow protein. Biochemistry.

[8] Kamikubo H (2007). Characterization of the solution structure of the M intermediate of photoactive yellow protein using high-angle solution x-ray scattering. Biophys. J..

[9] Shimizu N (2006). pH-Dependent equilibrium between long lived near-UV intermediates of photoactive yellow protein. J. Biol. Chem..

[10] Imamoto Y (1997). Evidence for proton transfer from Glu 46 to the chromophore during the photocycle of photoactive yellow protein. J. Biol. Chem..

[11] Borgstahl GE, Williams DR, Getzoff ED (1995). 1.4 Å structure of photoactive yellow protein, a cytosolic photoreceptor: unusual fold, active site, and chromophore. Biochemistry.

[12] Blakeley MP, Hasnain SS, Antonyuk SV (2015). Sub-atomic resolution X-ray crystallography and neutron crystallography: Promise, challenges and potential. IUCrJ.

[13] Cleland WW, Kreevoy MM (1994). Low-Barrier Hydrogen Bonds and Enzymaic Catalysis. Science.

[14] Frey PA, Whitt SA, Tobin JB (1994). A low-barrier hydrogen bond in the catalytic triad of serine proteases. Science.

[15] Saito K, Ishikita H (2012). Energetics of short hydrogen bonds in photoactive yellow protein. Proc. Natl. Acad. Sci. USA..

[16] Hirano K, Sato H (2013). A theoretical study on the electronic structure of PYP chromophore in low barrier hydrogen bonding model. Chem. Phys..

[17] Saito K, Ishikita H (2012). H atom positions and NMR chemical shifts of short H bonds in photoactive yellow protein. Biochemistry.

[18] Saito K, Ishikita H (2013). Formation of an unusually short hydrogen bond in photoactive yellow protein. Biochim. Biophys. Acta Bioenerg.

[19] Ishikita H, Saito K (2014). Proton transfer reactions and hydrogen-bond networks in Proton transfer reactions and hydrogen- bond networks in protein environments. J. R. Soc. Interface.

[20] Kanematsu Y, Tachikawa M (2014). Theoretical analysis of geometry and NMR isotope shift in hydrogen-bonding center of photoactive yellow protein by combination of multicomponent quantum mechanics and ONIOM scheme. J. Chem. Phys..

[21] Tamura K, Hayashi S (2015). Role of bulk water environment in regulation of functional hydrogen-bond network in photoactive yellow protein. J. Phys. Chem. B.

[22] Kanematsu Y, Kamikubo H, Kataoka M, Tachikawa M (2016). Vibrational analysis on the revised potential energy curve of the low-barrier hydrogen bond in photoactive yellow protein. Comput. Struct. Biotechnol. J..

[23] Nadal-ferret M, Gelabert R, Moreno M (2014). Are there really low-barrier hydrogen bonds in proteins? The case of photoactive yellow protein. J. Am. Chem. Soc..

[24] Adams PD, Mustyakimov M, Afonine PV, Langan P (2009). Generalized X-ray and neutron crystallographic analysis: More accurate and complete structures for biological macromolecules. Acta Crystallogr. Sect. D Biol. Crystallogr..

[25] Adams PD (2010). PHENIX: A comprehensive Python-based system for macromolecular structure solution. Acta Crystallogr. Sect. D Biol. Crystallogr..

[26] Afonine PV (2010). Joint X-ray and neutron refinement with phenix.refine. Acta Crystallogr. Sect. D Biol. Crystallogr..

[27] Anderson S, Crosson S, Moffat K (2004). Short hydrogen bonds in photoactive yellow protein. Acta Crystallogr. Sect. D Biol. Crystallogr..

[28] Sugishima M (2004). Structure of photoactive yellow protein (PYP) E46Q mutant at 1.2 Å resolution suggests how Glu46 controls the spectroscopic and kinetic characteristics of PYP. Acta Crystallogr. Sect. D Biol. Crystallogr..

[29] Schotte F (2012). Watching a signaling protein function in real time via 100-ps time-resolved Laue crystallography. Proc. Natl. Acad. Sci. USA..

[30] Genick UK (1997). Structure of a Protein Photocycle Intermediate by Millisecond Time – Resolved Crystallography. Science.

[31] Yamaguchi S (2007). Preparation of large crystals of photoactive yellow protein for neutron diffraction and high resolution crystal structure analysis. Photochem. Photobiol..

[32] Mihara K, Hisatomi O, Imamoto Y, Kataoka M, Tokunaga F (1997). Functional expression and site-directed mutagenesis of photoactive yellow protein. J. Biochem..

[33] Kurihara K, Tanaka I, Muslih MR, Ostermann A, Niimura N (2004). A new neutron single crystal diffractometer dedicated for biological macromolecules (BIX-4). J. Synchrotron Radiat..

[34] Otwinowski Z, Minor W (1997). Processing of X-ray diffraction data collected in oscil- lation mode. Methods Enzymol..

[35] McRee DE (1999). XtalView/Xfit-A versatile program for manipulating atomic coordinates and electron density. J. Struct. Biol..

[36] Emsley P, Cowtan K (2004). Coot: Model-building tools for molecular graphics. Acta Crystallogr. Sect. D Biol. Crystallogr..

[37] Zoete V, Cuendet MA, Grosdidier A, Michielin O (2011). SwissParam: A fast force field generation tool for small organic molecules. J. Comput. Chem..

[38] Delano, W. L. *The PyMOL Molecular Graphics System* (2002).

[39] Sheldrick GM, Schneider TR (1997). SHELXL: High-resolution refinement. Methods Enzymol..

[40] Gruene T, Hahn HW, Luebben AV, Meilleur F, Sheldrick GM (2014). Refinement of macromolecular structures against neutron data with SHELXL2013. J. Appl. Crystallogr..

