# Supplementary material for: Neutron crystallography of photoactive yellow protein reveals unusual protonation state of Arg52 in the crystal
Source: Sci Rep. 2017 Aug 24;7:9361. doi: 10.1038/s41598-017-09718-9 (PMC5570954; doi:10.1038/s41598-017-09718-9)
Supplement: Supplementary file 1 — Supplementary Information [file 41598_2017_9718_MOESM1_ESM.pdf]

Supplementary Information for

Neutron crystallography of photoactive yellow protein reveals unusual protonation state of Arg52 in the crystal

Kento Yonezawa, Nobutaka Shimizu, Kazuo Kurihara, Yoichi Yamazaki,

Hironari Kamikubo, & Mikio Kataoka

Supplemental Figures

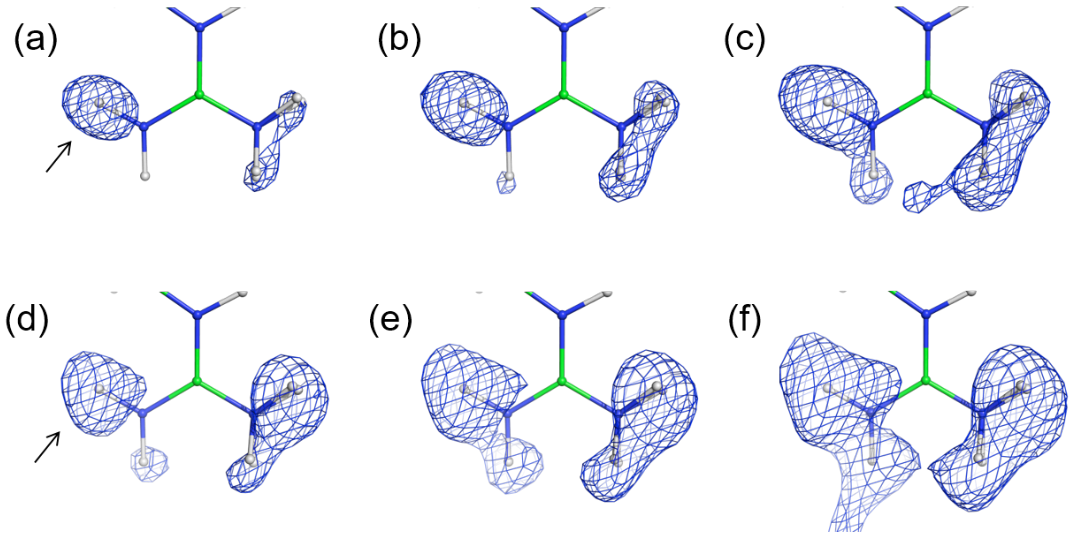

Supplementary Figure 1. Fo–Fc Difference Fourier maps of the deuterium on the guanidino group of Arg52. (a)–(c) show the guanidino structures and nuclear densities of Arg52 in WT, and (d)–(f) show those in E46Q. Blue mesh represents positive nuclear densities of the deuterium atoms, contoured at (a) (d) 50%, (b) (e) 40%, and (c) (f) 30% of the maximum peak height of the Fo–Fc difference maps of the deuterium atom shown in (a) for WT and (e) for E46Q (arrows); contour levels of maximum peak height correspond to 11.4σ for WT and 10.0σ for E46Q.

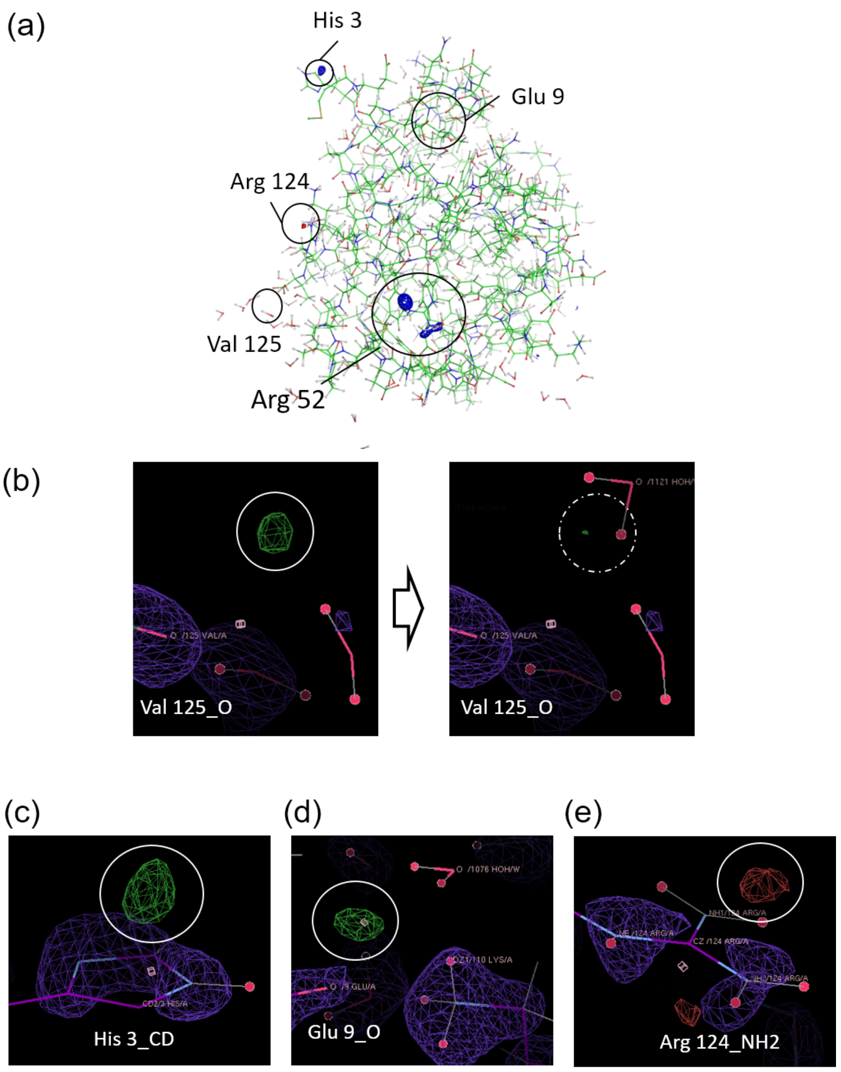

Supplementary Figure 2. Fo–Fc Difference Fourier maps of WT. (a) Fo–Fc Difference Fourier map at the counter level of +5.5 σ. The peaks above +6 σ level are circuled. (b)–(e) Enlarged view of each peak. The nuclear density in (b) can be removed by adding a water molecule. The origin of the rest of three (c-e) are still unclear, but they locate distant from the Arg52 we focus.

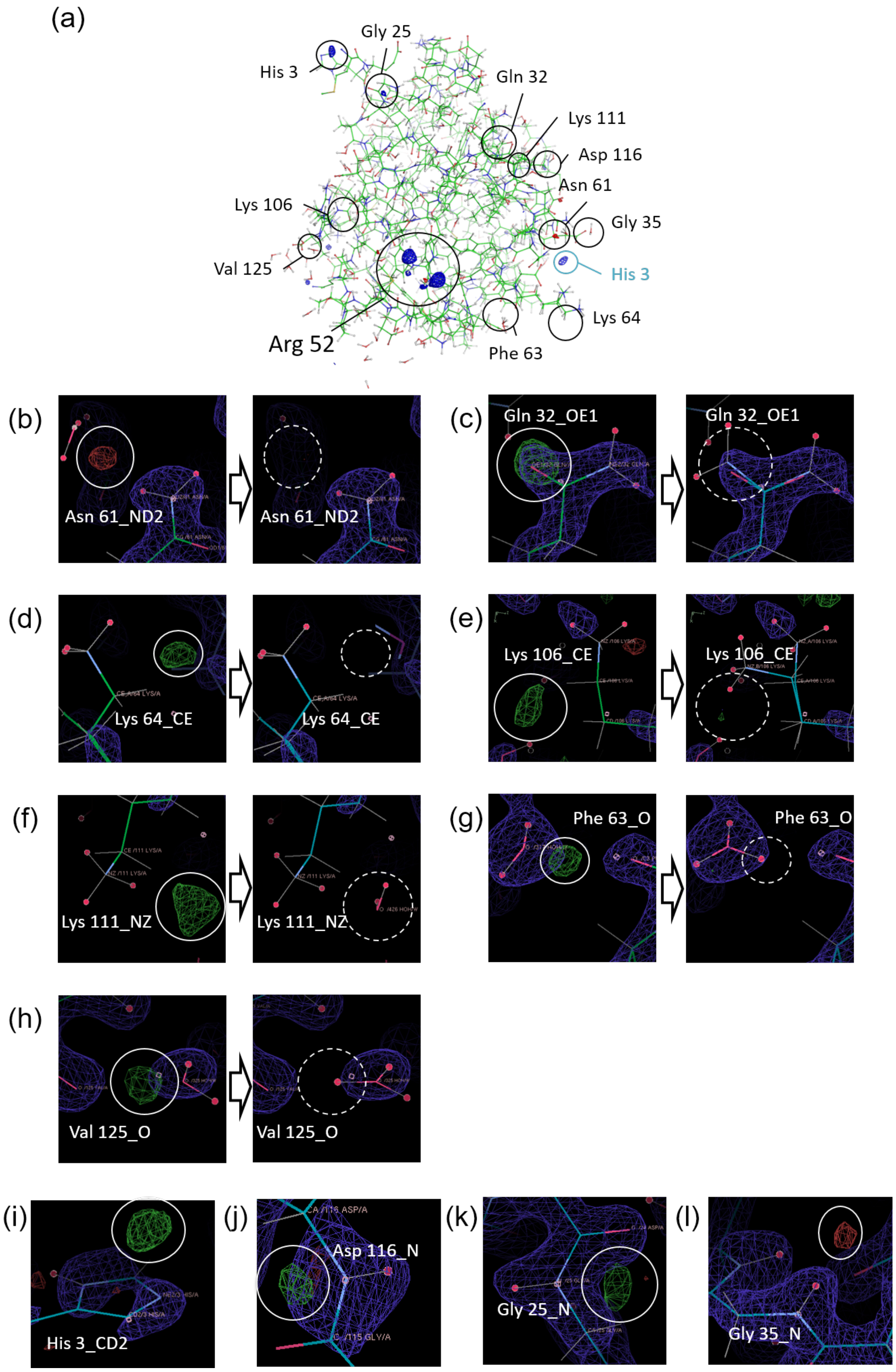

Supplementary Figure 3. Fo–Fc Difference Fourier maps of WT. (a) Fo–Fc Difference Fourier map at the counter level of +5.5 σ. The peaks above +6 σ level are circuled. (b)–(l) Enlarged view of each peak. The nuclear densities in (b-h) can be removed by adding a water molecule or an alternative structure. The origin of the rest of four (i-l) are still unclear, but they locate distant from the Arg52 we focus.

Supplementary Table 1. Hydrogen bond lengths (Å) and interatomic distances (Å) near the chromophore. The estimated standard deviation values of the hydrogen bond lengths calculated by shelxL2013 are shown in parentheses.

	pCA-Glu46	Glu46-O…D	D… O-pCA
Current WT	2.55 (0.03)	1.26	1.35
WT (2ZOI)	2.56 (0.03)	1.21	1.37
E46Q	2.85 (0.04)	1.02	1.84

	pCA-Tyr42	Tyr42-O-D	D… O-Tyr42
Current WT	2.53 (0.03)	0.97	1.61
WT (2ZOI)	2.54 (0.03)	0.96	1.65
E46Q	2.46 (0.04)	0.97	1.56

Supplementary Table 2. Refinement parameters of deuterium on guanidino group of Arg52. (a) WT (b) E46Q.

	Atom	Occupancy	*B*-factor
(a)	D_{11}	1	12.51
	D_{12}	0.24	9.4
	$D_{21}(sp^2)$		17.09
	$D_{22}(sp^2)$		21.06
	$D_{21}(sp^3)$ upward	0.40	15.19
	$D_{22}(sp^3)$ upward		15.29
	$D_{21}(sp^3)$ downward	0.36	25.15
	$D_{22}(sp^3)$ downward		22.57
(b)	D_{11}	1	18.41
	D_{12}	0.67	24.4
	$D_{21}(sp^2)$		17.28
	$D_{22}(sp^2)$		29.75
	$D_{21}(sp^3)$	0.33	19.21
	$D_{22}(sp^3)$		28.18

Supplementary Table 3. X-ray and neutron diffraction and refinement data statistics of the wild type.

	Neutron	X-ray
Data collection statics		
Space group	$P6_3$	$P6_3$
Unit-cell parameters		
a, b, c (Å)	67.15, 67.15, 41.12	66.83, 66.83, 40.92
α, β, γ (°)	90.0, 90.0, 120.0	90.0, 90.0, 120.0
Resolution (Å)	100.00 – 1.50 (1.55–1.50)	57.83 – 1.25 (1.32–1.25)
R_{merge} (%)	10.9 (32.4)	5.8 (35.6)
$< I / \sigma(I) >$	7.4 (2.9)	21.9 (7.3)
Completeness (%)	89.6 (72.8)	100 (100)
Redundancy	2.6 (2.0)	11.1 (11.3)
Unique reflections	15,283	28,951
	Neutron / X-ray joint refinement	
Resolution (Å)	33.41 – 1.49	33.42 – 1.25
R_{work}/R_{free} (%)	17.2 / 19.9	14.8 / 15.36
	Neutron refinement	
Resolution (Å)	33.44 – 1.49	
R_{work}/R_{free} (%)	16.2 / 20.8	
Average B factors (Å^2)		
Protein	16.2	
Ligand/ion	7.22	
Water	41.9	
R.m,s.d.		
Bond lengths (Å)	0.007	
Bond angles (°)	1.205	

Highest-resolution shell is shown in parentheses.

Supplementary Table 4. X-ray and neutron diffraction and refinement data statistics of the E46Q mutant.

	Neutron	X-ray
Data collection statics		
Space group	$P6_3$	$P6_3$
Unit-cell parameters		
a, b, c (Å)	67.14, 67.14, 41.17	66.82, 66.82, 40.98
α, β, γ (°)	90.0, 90.0, 120.0	90.0, 90.0, 120.0
Resolution (Å)	100.00 – 1.50 (1.55–1.50)	50.00 – 1.30 (1.32–1.30)
R_{merge} (%)	9.5 (31.8)	5.0 (31.0)
$< I / \sigma(I) >$	7.78 (2.44)	130.2 (16.5)
Completeness (%)	93.4 (85.7)	98.8 (96.8)
Redundancy	2.2 (1.6)	55.5 (55.7)
Unique reflections	15,985	25,504
	Neutron / X-ray joint refinement	
Resolution (Å)	33.44 – 1.49	33.45 – 1.30
R_{work}/R_{free} (%)	16.8 / 19.5	14.8 / 15.8
	Neutron refinement	
Resolution (Å)	33.44 – 1.49	
R_{work}/R_{free} (%)	15.8/20.0	
Average B factors (Å^2)		
Protein	16	
Ligand/ion	9.22	
Water	33.74	
R.m,s.d.		
Bond lengths (Å)	0.009	
Bond angles (°)	1.15	

Highest-resolution shell is shown in parentheses.
